# Tolerance to Gamma Radiation in the Marine Heterotardigrade, *Echiniscoides sigismundi*

**DOI:** 10.1371/journal.pone.0168884

**Published:** 2016-12-20

**Authors:** K. Ingemar Jönsson, Thomas L. Hygum, Kasper N. Andersen, Lykke K. B. Clausen, Nadja Møbjerg

**Affiliations:** 1 School of Education and Environment, Kristianstad University, Kristianstad, Sweden; 2 Department of Biology, University of Copenhagen, Copenhagen, Denmark; Mizoram University, INDIA

## Abstract

Tardigrades belong to the most radiation tolerant animals on Earth, as documented by a number of studies using both low-LET and high-LET ionizing radiation. Previous studies have focused on semi-terrestrial species, which are also very tolerant to desiccation. The predominant view on the reason for the high radiation tolerance among these semi-terrestrial species is that it relies on molecular mechanisms that evolved as adaptations for surviving dehydration. In this study we report the first study on radiation tolerance in a marine tardigrade, *Echiniscoides sigismundi*. Adult specimens in the hydrated active state were exposed to doses of gamma radiation from 100 to 5000 Gy. The results showed little effect of radiation at 100 and 500 Gy but a clear decline in activity at 1000 Gy and higher. The highest dose survived was 4000 Gy, at which ca. 8% of the tardigrades were active 7 days after irradiation. LD50 in the first 7 days after irradiation was in the range of 1100–1600 Gy. Compared to previous studies on radiation tolerance in semi-terrestrial and limnic tardigrades, *Echiniscoides sigismundi* seems to have a lower tolerance. However, the species still fits into the category of tardigrades that have high tolerance to both desiccation and radiation, supporting the hypothesis that radiation tolerance is a by-product of adaptive mechanisms to survive desiccation. More studies on radiation tolerance in tardigrade species adapted to permanently wet conditions, both marine and freshwater, are needed to obtain a more comprehensive picture of the patterns of radiation tolerance.

## Introduction

Tardigrades are small invertebrate animals well-known for their ability to tolerate complete desiccation and very high and low temperatures [1,2,3]. They also belong to the most radiation tolerant animals on Earth and several studies have documented a very high tolerance of adult tardigrades to both low-LET (Linear Energy Transfer) radiation (X-rays: [4]; gamma rays: [5,6,7]), high-LET radiation (alpha particles: [6]; protons: [8]), and UV radiation [9,10]. Tardigrades are also the only animals so far that have survived the combined exposure to cosmic radiation, UV radiation and vacuum under real space conditions [11]. The dose-responses documented in these studies show that several tardigrade species are able to survive several thousands of Gray of ionizing radiation. For comparison, the dose at which 50% of humans die within 30 days is less than 5 Gy [12]. It is also clear that the tolerance is not restricted to the desiccated anhydrobiotic state in tardigrades, but is expressed also in active hydrated animals. Embryos are considerably more sensitive to radiation, particularly in the early stage of development [7,13,14,15].

The majority of previous studies on radiation tolerance in tardigrades have investigated limno-terrestrial species that are also highly tolerant to desiccation (*Richtersius coronifer*, *Milnesium tardigradum*, *Ramazzottius varieornatus*), the only exception being a study on the limnic species *Hypsibius dujardini* [7]. A common view on why tardigrades are highly tolerant to radiation is that their tolerance relies on molecular mechanisms that have evolved to allow survival under extreme but natural environmental conditions, in dry or cold environments [16]. This hypothesis predicts that the most desiccation tolerant tardigrades should also be the most radiation tolerant, regardless of evolutionary origin and taxonomic affiliation. So far, all tardigrades evaluated for radiation tolerance belong to the class of Eutardigrada. Here we report the first study on tolerance to ionizing radiation in a marine heterotardigrade species, *Echiniscoides sigismundi*, and evaluate the dose-response to gamma irradiation.

## Methods

### Collection and sorting of tardigrades

Specimens of the marine tidal heterotardigrade *Echiniscoides sigismundi* (Schultze, 1865) were collected from barnacle shells at Lynæs, Zealand, Denmark (55° 44' 08.10'' N, 11° 51' 27.23'' E). At the time of sampling on the 7^th^ of September 2015, the water at Lynæs had a measured salinity of 17.5 ‰ and a temperature of 17.3°C. The tardigrades were retrieved from the barnacle shells by freshwater-shocking and stored with substrate refrigerated in locality seawater for one week. One to two days prior to experimentation, the tardigrades were carefully cleaned and transferred into 27 Eppendorf tubes (2 mL) with approximately 20 animals in each. The tubes were filled with ca. 1 mL of filtered seawater from the locality.

### Experimental set-up and radiation source

The Eppendorf tubes with *Echiniscoides sigismundi* in seawater were transported from Copenhagen to Stockholm University (Department of Molecular Biology, Wenner-Gren Institute) by train (ca. 5h travel) for irradiation in the same day (starting at ca. 5 PM). During transport the samples were kept at low temperature in a cooling box (temp. range during travels 7–15°C), but were placed at ambient room temperature (ca. 23°C) about 1h before the first samples were irradiated. Samples were irradiated with gamma rays in a Gammacell 1000 ^137^Cs source (Isomedix, Inc., Kanata, Ontario, Canada) at a dose rate of 6.22 Gy/min. Ambient temperature in the room with the irradiation source was measured at ca. 23°C. From the start to the end of the irradiation period (05:06 PM– 06:30 AM), all samples were kept at room temperature. Three replicate tubes were irradiated for each of the following doses: 100, 500, 1000, 2000, 3000, 4000 and 5000 Gy. Total irradiation time ranged from 16.1 minutes for the 100 Gy samples to 13h 24 min for the 5000 Gy samples. After irradiation new filtered seawater was added to the samples. After all samples were irradiated they were again kept under cooled condition in a cooling box, and immediately transported by train to Copenhagen. Three sample tubes were kept under the same conditions as the irradiated samples, but were not irradiated, thus representing controls.

After their return to Copenhagen, the tardigrades were transferred to 12-well cell culturing plates and were left refrigerated in these plates for the subsequent assessment period. The activity assessments were performed under microscope at room temperature.

### Scoring of activity

The activity level of single specimens was recorded by observing the tardigrades under a stereomicroscope (Zeiss Stemi 2000) at 40-50X magnification. The tardigrades were considered active if they showed clear spontaneous movements of legs or the main body or were responsive to a tactile stimulus (using a small syringe). All animals entering the experiment showed clear activity prior to experimentation. As inactive tardigrades are not necessarily dead, the activity data reported represent an underestimate of survival rates. The specimens were monitored for a period of 7 days, with activity cheeks 24h, 48h, 72h and 7 days post-irradiation.

### Statistical analyses and dose response curve fitting

We used the Jonckheere-Terpstra nonparametric test for ordered alternatives to test the hypothesis that tardigrade activity declined with higher dose (overall and pairwise comparisons), and the Kruskal-Wallis Analysis of Variance to test for differences in activity between activity estimates within dose-groups. Statistical results were considered significant when P<0.05. One-tailed probabilities are reported from the Jonckheere-Terpstra test (negative dose-dependence expected), and two-tailed probabilities from the Kruskal-Wallis tests (no specific trend expected). Complete statistical results are provided in the Supporting Information File (S1 Appendix). Dose-response curves and LD50-values were obtained from the software OriginPro 9.1 (OriginLab, Northampton, Massachusetts, USA).

## Results

**Fig 1:**
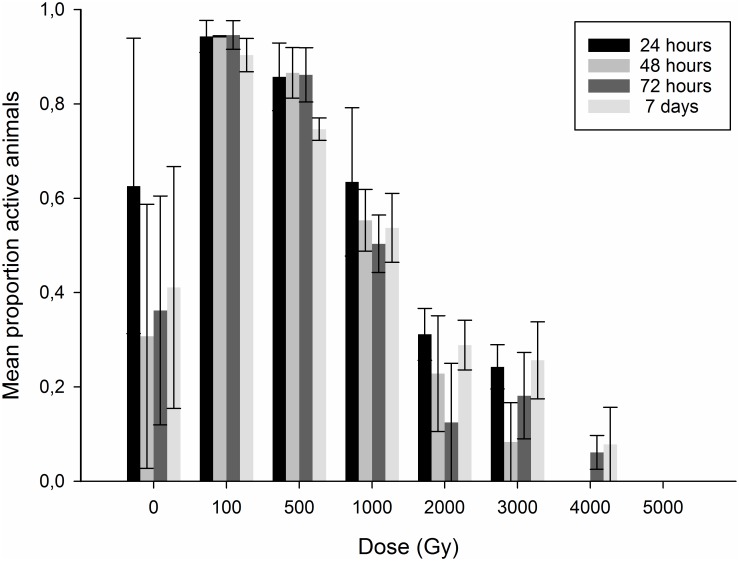
Mean proportion of adult *Echiniscoides sigismundi* showing activity after exposure to different doses of gamma radiation. The figure shows estimates from four different time points after irradiation, from 24h to 7 days. Error bars represent standard errors of the mean from 3 repeats, each with approx. 20 animals.

The non-irradiated samples showed a strong deviation in activity scores compared to the samples irradiated at the lowest doses (Fig 1), with one replicate sample containing no active specimens and another with much reduced activity levels (6–35%) after the 24 hour estimate. Obviously, some factor (see Discussion) affected the non-irradiated samples, and we therefore excluded these in the analysis below.

No statistical differences in the percentage of active animals were found among the 24h, 48h, 72h and 7 day post-irradiation estimates within each dose, while the estimated activity generally declined significantly with higher dose (P<0.0025 for all overall estimates; Fig 1, S1 Appendix). For pairwise comparisons among dose-groups the pattern remained relatively similar from the 24h to the 7 day estimate, but with less significant differences among the lowest dose groups and more differences among the highest dose groups at the 24h estimate compared to later estimates (S1 Appendix). Average activity varied between 90% and 95% over the 7 day period for the 100 Gy group. In contrast, the highest doses (2–5 kGy) in all except one case had significantly lower activity compared to the lower doses (100 Gy–1 kGy). In the 4 kGy group no activity was recorded in the first two evaluations, but active animals appeared in the 72h and 7 days evaluations. Average activity after 7 days in this dose group was 7.8%, but only one of the three replicate samples contained active animals. No animal showed signs of activity over 4 kGy.

**Fig 2:**
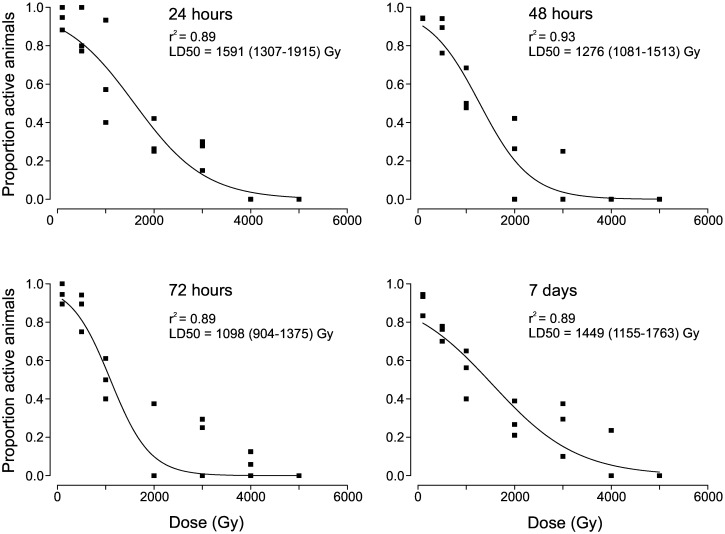
Dose-response curves showing the proportion of active *Echiniscoides sigismundi* after different doses of gamma radiation, at activity assessments 24h, 48h, 72h and 7 days after irradiation. Data for non-irradiated samples were excluded. Each data point shows the proportion of active tardigrades out of approx. 20 animals. LD50 values (with 95% confidence intervals in parentheses) obtained from dose-response fitting and the statistical support of the regressions are given within each graph.

Dose-response curves were fitted for data from all four evaluation times, and resulted in LD50 values between 1098 Gy and 1591 Gy, with the highest values at the 24h and 7 days estimates (Fig 2). The curves expressed weak sigmoidal shapes with no clear shoulders.

## Discussion

Our study provides the first evidence that tardigrades also of the class Heterotardigrada have a high tolerance to ionizing radiation, with some specimens surviving doses of gamma ray up to 4 kGy. High radiation tolerance therefore is not restricted to the class Eutardigrada but seems to have a wide taxonomic distribution within the phylum Tardigrada. However, in contrast to previous dose-response studies with gamma rays in eutardigrades, which exhibit LD50 values in the range of 3–5 kGy, 1–2 days after irradiation [5,6,7], calculated LD50 values of *E*. *sigismundi* were considerably lower. Already at 1 kGy the effect of irradiation on animal activity was considerable, while in the eutardigrades *Macrobiotus areolatus* [4], *Richtersius coronifer* [5], *Milnesium tardigradum* [6], and *Hypsibius dujardini* [7], doses of 3 kGy or higher were required to significantly reduce viability. Thus, under the current protocol, *E*. *sigismundi* seems to be more sensitive to low-LET ionizing radiation than the eutardigrades studied so far.

The mechanisms behind the unnaturally high tolerance to radiation seen in tardigrades have not been revealed, but a functional connection with adaptive mechanisms evolved to allow survival in naturally dry environmental conditions has been suggested [16]. This explanation has been suggested also for other desiccation tolerant organisms with high radiation tolerance, such as the bacterium *Deinococcus radiodurans* [17], the chironomid insect *Polypedilum vanderplanki* [18] and bdelloid rotifers [19]. Radiation tolerance would then be a by-product of tolerance to desiccation, rather than an adaptation *per se*. If this hypothesis is correct species with high radiation tolerance would also be expected to have high desiccation tolerance, and all except one of the above mentioned species fit well into this picture. The exception is the freshwater tardigrade *H*. *dujardini*, which has a considerably lower tolerance to desiccation than the other species [20,10], but still shows very high tolerance to radiation [7]. The results from the current study on *E*. *sigismundi* tend to fit relatively well into the hypothesis. *E*. *sigismundi* is a marine species adapted to tidal habitats with recurrent exposure to dry conditions on barnacle shells, and with a recently documented ability to tolerate complete desiccation (i.e., anhydrobiosis) [21,22]. In contrast to limno-terrestrial tardigrades it can survive desiccation with or without contracting into a so-called “tun” [22]. Thus it must clearly be considered a desiccation-tolerant tardigrade. However, the ability of *E*. *sigismundi* to survive long periods in the anhydrobiotic state or to tolerate different rates of desiccation has not been studied. Although its tolerance to gamma radiation seems to be lower than in the eutardigrades previously tested, an LD50 > 1 kGy is still very high and places this species towards the high desiccation—high radiation tolerance area.

To obtain a better view of the extent to which desiccation tolerance and radiation tolerance are linked, more studies on radiation tolerance in less desiccation tolerant species are needed. Studies of other marine and freshwater tardigrade species adapted to more permanently wet conditions are of great interest in this context. It would also be of interest to investigate if the limno-terrestrial species of the heterotardigrade order Echiniscoidea exhibit a high tolerance to radiation, since many of them (if not all) are desiccation tolerant.

The evaluation of the hypothesis of a direct connection between desiccation and radiation tolerance is complicated by the fact that desiccation tolerance is not a straightforward concept, but includes several aspects that should be taken into account. Such aspects include the *rate of dehydration*, the *level of dehydration* and the *length of time* in the desiccated state that a given organism tolerates. These aspects of adaptation are expected to be strongly related to the environment in which the organism evolved, and the underlying mechanisms for desiccation tolerance may not necessarily be identical. For instance, morphological, physiological and biochemical mechanisms underlying the ability to survive rapid desiccation and long-term desiccation, respectively, may be different, and this could also influence the link between desiccation and radiation tolerance in a specific species. *E*. *sigismundi* tolerates severe desiccation, but its ability to stay in the dry state over longer time periods remains to be documented. The short-term change in water level characterizing tidal habitats predicts that it is normally exposed to short periods of desiccation. It could, therefore, be speculated that its lower tolerance to radiation compared to the limno-terrestrial eutardigrade species may be related to a lower tolerance for long-term anhydrobiosis. On the other hand, the tidal habitat may, during winter months, be subjected to prolonged periods of subzero temperatures imposing extracellular freezing of tardigrade body fluids and thus severe cellular dehydration. Thus strong mechanisms for cellular and molecular stabilization and/or repair should be in place in this species as they are in anhydrobiotic limno-terrestrial eutardigrades [e.g. 23]. The underlying mechanisms could, however, differ between the different types of adaptations as well as between *tardigrade lineages* [24], underlining the importance of investigations into more heterotardigrade species.

Apart from the question of a connection between desiccation and radiation tolerance, it should be considered that *E*. *sigismundi* is a marine invertebrate, and that the animals were kept in their natural sea water during irradiation. Marine invertebrates are not considered to deviate in sensitivity and response to ionizing radiation compared to freshwater invertebrates [25], but few comparative studies are available. There are also few studies on radiation tolerance in desiccation tolerant marine invertebrates. A notable exception is the brine shrimp *Artemia*, which inhabits hypersaline biotypes worldwide, and in its embryonic (cyst) stage is extremely tolerant to both desiccation [26] and ionizing radiation [27]. Concerning the potential importance of water salinity for the irradiation response, Dallas et al. [28] reviewed some examples of interactions between salinity level (of irradiation medium) and irradiation response in some marine crustaceans (including *Artemia*), and suggested that “in addition to salinity affecting radiation tolerance, radiation also interferes with the capacity of these organisms to deal with changes in salinity.” Whether the lower tolerance to radiation (compared to semi-terrestrial species) found in *E*. *sigismundi* is somehow related to the saline environment remains to be studied.

Three non-irradiated samples of tardigrades were included in the current protocol as controls. These specimens were treated similarly to the irradiated samples, but nevertheless had a much lower and more variable activity than the low dose irradiated groups. It is our experience that *Echiniscoides sigismundi* is sensitive to water contaminants. As irradiation likely killed microorganisms present in the sea water, one possible explanation, for the discrepancy in activity between non-irradiated and low-dose irradiated samples, could be that microorganisms affected the non-irradiated samples negatively. In future experiments where natural sea water has to be used, transfer of control samples into irradiated sea water could provide a solution to this potential problem.

The appearance of active tardigrades in the 4 kGy dose-group only after 72 hours is notable, and although not statistically significant a similar delay in activity was observed also in the 2 and 3 kGy dose-groups. Delays in recovery has been reported in tardigrades after long-term desiccation [29,30], but similar patterns related to radiation dose has not previously been indicated for adult tardigrades. However, Beltrán-Pardo et al. [14] reported a dose-related delay in egg development in *M*. *tardigradum* after irradiation with gamma ray. Such delays are generally interpreted as indicating cell cycle arrest and the activity of repair processes.

In conclusion, the current study broadens our knowledge on radiation tolerance in tardigrades to include also the marine heterotardigrade *E*. *sigismundi*. An inclusion of non-tidal marine tardigrade species and limno-terrestrial heterotardigrades in future studies would further contribute to the picture of the taxonomic and environmental distribution of radiation tolerance in tardigrades.

## Supporting Information

S1 Appendix Statistical results related to analyses in the article. (DOCX)
