# Supplementary material for: Tolerance to Gamma Radiation in the Marine Heterotardigrade, Echiniscoides sigismundi
Source: PLoS One. 2016 Dec 20;11(12):e0168884. doi: 10.1371/journal.pone.0168884 (PMC5173286; doi:10.1371/journal.pone.0168884)
Supplement: S1 Appendix — (DOCX) [file pone.0168884.s001.docx]

**S1 Appendix. Statistical results related to figure 1 in Jönsson et al. 2016.**

**1. Overall and post hoc analyses of statistical trend (negative dose-dependence) in activity among dose-groups of *Echinicoides sigismundi* exposed to gamma radiation, using the Jonckheere-Terpstra nonparametric test for ordinal independent variables.** Significant p-values < 0.05 are marked in red. For both overall and pairwise analyses one-tailed p-values are presented. Note that non-irradiated control samples were not included in this analysis due to unexpectedly low activity (see Discussion for the possible cause of this effect).

**Table S1-1.** P-values for the 24h post-irradiation estimate data. Overall Jonckheere-Terpstra analysis: T_JT_ = 15.0, *z* = –4.92, *p* < 0.00025.

| **Dose (Gy)** | **100** | **500** | **1000** | **2000** | **3000** | **4000** | **5000** |
| --- | --- | --- | --- | --- | --- | --- | --- |
| **100** | - | 0.19 | 0.063 | 0.025 | 0.025 | 0.018 | 0.018 |
| **500** | - | - | 0.14 | 0.025 | 0.025 | 0.018 | 0.018 |
| **1000** | - | - | - | 0.063 | 0.025 | 0.018 | 0.018 |
| **2000** | - | - | - | - | 0.41 | 0.018 | 0.018 |
| **3000** | - | - | - | - | - | 0.018 | 0.018 |
| **4000** | - | - | - | - | - | - | 0.50 |
| **5000** | - | - | - | - | - | - | - |

**Table S1-2.** P-values for the 48h post-irradiation estimate data. Overall Jonckheere-Terpstra analysis: T_JT_ = 16.0, *z* = –5.01, *p* < 0.00025.

| **Dose (Gy)** | **100** | **500** | **1000** | **2000** | **3000** | **4000** | **5000** |
| --- | --- | --- | --- | --- | --- | --- | --- |
| **100** | - | 0.036 | 0.023 | 0.023 | 0.022 | 0.017 | 0.017 |
| **500** | - | - | 0.025 | 0.025 | 0.023 | 0.018 | 0.018 |
| **1000** | - | - | - | 0.025 | 0.023 | 0.018 | 0.018 |
| **2000** | - | - | - | - | 0.12 | 0.061 | 0.061 |
| **3000** | - | - | - | - | - | 0.16 | 0.16 |
| **4000** | - | - | - | - | - | - | 0.50 |
| **5000** | - | - | - | - | - | - | - |

**Table S1-3.** P-values for the 72h post-irradiation estimate data. Overall Jonckheere-Terpstra analysis: T_JT_ = 20.0, *z* = –4.65, *p* < 0.00025.

| **Dose (Gy)** | **100** | **500** | **1000** | **2000** | **3000** | **4000** | **5000** |
| --- | --- | --- | --- | --- | --- | --- | --- |
| **100** | - | 0.092 | 0.025 | 0.023 | 0.025 | 0.025 | 0.018 |
| **500** | - | - | 0.025 | 0.023 | 0.025 | 0.025 | 0.018 |
| **1000** | - | - | - | 0.023 | 0.025 | 0.025 | 0.018 |
| **2000** | - | - | - | - | 0.41 | 0.41 | 0.16 |
| **3000** | - | - | - | - | - | 0.19 | 0.061 |
| **4000** | - | - | - | - | - | - | 0.061 |
| **5000** | - | - | - | - | - | - | - |

**Table S1-4.** P-values for the 7 days post-irradiation estimate data. Overall Jonckheere-Terpstra analysis: T_JT_ = 9.0, *z* = –5.26, *p* < 0.00025.

| **Dose (Gy)** | **100** | **500** | **1000** | **2000** | **3000** | **4000** | **5000** |
| --- | --- | --- | --- | --- | --- | --- | --- |
| **100** | - | 0.025 | 0.025 | 0.025 | 0.025 | 0.023 | 0.018 |
| **500** | - | - | 0.025 | 0.025 | 0.025 | 0.023 | 0.018 |
| **1000** | - | - | - | 0.025 | 0.025 | 0.023 | 0.018 |
| **2000** | - | - | - | - | 0.41 | 0.061 | 0.018 |
| **3000** | - | - | - | - | - | 0.061 | 0.018 |
| **4000** | - | - | - | - | - | - | 0.16 |
| **5000** | - | - | - | - | - | - | - |

**2. Kruskal-Wallis Analysis of Variance tests for overall differences in activity between activity estimates (24h, 48h, 72h, 7 days) within dose-groups.**

100 Gy: χ^2^ = 1.91, df = 3, P = 0.59.

500 Gy: χ^2^ = 2.92, df = 3, P = 0.41.

1000 Gy: χ^2^ = 0.43, df = 3, P = 0.93.

2000 Gy: χ^2^ = 3.28, df = 3, P = 0.28.

3000 Gy: χ^2^ = 3.65, df = 3, P = 0.30.

4000 Gy: χ^2^ = 3.93, df = 3, P = 0.27.
